# Improvement of pain and regional osteoporotic changes in the foot and ankle by low-dose bisphosphonate therapy for complex regional pain syndrome type I: a case series

**DOI:** 10.1186/1752-1947-5-349

**Published:** 2011-08-04

**Authors:** Yasuhisa Abe, Kousuke Iba, Junichi Takada, Takuro Wada, Toshihiko Yamashita

**Affiliations:** 1 Department of Orthopedic Surgery, Sapporo Medical University School of Medicine, Sapporo 060-8543, Japan; 2 Kitago Orthopedic Clinic, Sapporo, Japan

## Abstract

**Introduction:**

Complex regional pain syndrome is characterized by pain, allodynia, hyperalgesia, edema, signs of vasomotor instability, movement disorders, joint stiffness, and regional osteopenia. It is recognized to be difficult to treat, despite various methods of treatment, including physiotherapy, calcitonin, corticosteroids, sympathetic blockade, and nonsteroidal anti-inflammatory drugs. Pathophysiologically, complex regional pain syndrome reveals enhanced regional bone resorption and high bone turnover, and so bisphosphonates, which have a potent inhibitory effect on bone resorption, were proposed for the treatment of complex regional pain syndrome.

**Case presentation:**

A 48-year-old Japanese man with complex regional pain syndrome type I had severe right ankle pain with a visual analog scale score of 59 out of 100 regardless of treatment with physiotherapy and nonsteroidal anti-inflammatory drugs for five months. Radiographs showed marked regional osteoporotic changes and bone scintigraphy revealed a marked increase in radioactivity in his ankle. One month after the start of oral administration of risedronate (2.5 mg per day), his bone pain had fallen from a VAS score of 59 out of 100 to 18 out of 100. Bone scintigraphy at 12 months showed a marked reduction in radioactivity to a level comparable to that in his normal, left ankle. On the basis of these results, the treatment was discontinued at 15 months. At 32 months, our patient had almost no pain and radiographic findings revealed that the regional osteoporotic change had returned to normal.

A second 48-year-old Japanese man with complex regional pain syndrome type I had severe right foot pain with a visual analog scale score of 83 out of 100 regardless of treatment with physiotherapy and nonsteroidal anti-inflammatory drugs for nine months. Radiographs showed regional osteoporotic change in his phalanges, metatarsals, and tarsals, and bone scintigraphy revealed a marked increase in radioactivity in his foot. One month after the start of oral administration of alendronate (35 mg per week), his bone pain had fallen from a visual analog scale score of 83 out of 100 to 30 out of 100 and, at nine months, was further reduced to 3 out of 100. The treatment was discontinued at 15 months because of successful pain reduction. At 30 months, our patient had no pain and the radiographic findings revealed marked improvement in regional osteoporotic changes.

**Conclusions:**

We believe low-dose oral administration of bisphosphonate is worth considering for the treatment of idiopathic complex regional pain syndrome type I accompanied by regional osteoporotic change.

## Introduction

Complex regional pain syndrome (CRPS) is characterized by pain, allodynia, hyperalgesia, edema, signs of vasomotor instability, movement disorders, joint stiffness, and regional osteopenia [1,2]. Various methods of treatment - including physiotherapy, calcitonin, corticosteroids, sympathetic blockade, and nonsteroidal anti-inflammatory drugs (NSAIDs) - have been tried [3-5]. However, in many cases, pain and the ensuing loss of function are permanent despite treatment [2,6]. On the other hand, previous studies reported that accelerated and enhanced bone resorption and turnover play a central pathophysiological role in CRPS [6,7]. Accordingly, bisphosphonates, which have a potent inhibitory effect on bone resorption, were proposed for the treatment of CRPS. In fact, several studies indicated that the intravenous or high-dose oral administration of bisphosphonate improved pain and reduced bone turnover in CRPS cases [8-11]. In this report, we present two cases of CRPS in which a low dose of oral risedronate (2.5 mg per day) or alendronate (35 mg per week) markedly decreased pain and regional osteoporotic findings in the foot or ankle. Also, since the discontinuation of the bisphosphonate treatment, our patients have not complained of bone pain, and normal bone turnover has been observed for more than one year of follow-up without additional treatment.

## Case presentation

**Figure 1:**
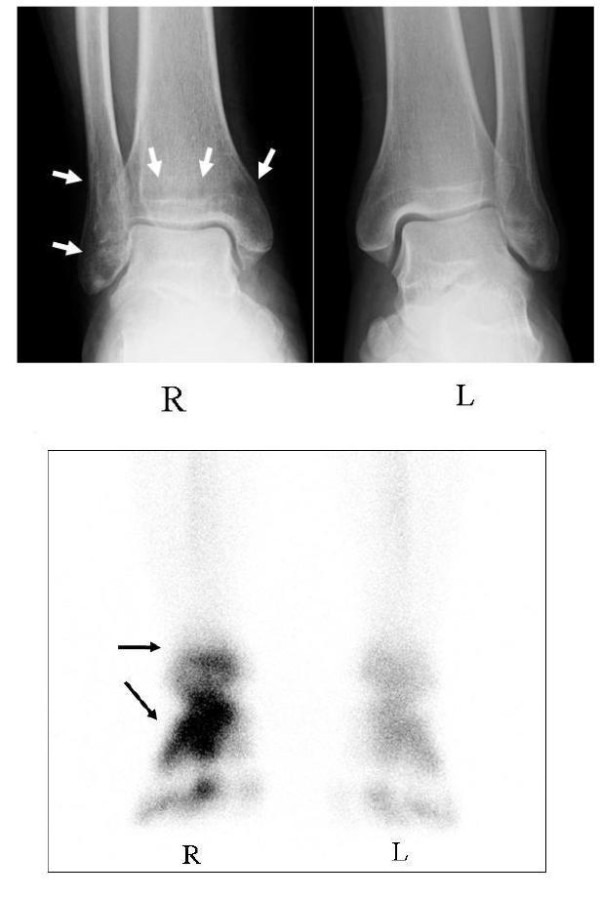
**Radiographs (A) and scintigraphs (B) of the both ankles before treatment**. The radiograph of the right ankle showed regional osteoporotic findings at the distal tibia and fibula (white arrow) (A). Bone scintigraphy with ^99 m^Tc-methylene diphosphonate showed a marked increase in radioactivity around the ankle (black arrow) (B).

A 48-year-old Japanese man with CRPS was referred to our institute for the treatment of severe right ankle pain with a visual analog scale (VAS) [12] score of 59 out of 100. About five months earlier, he began to feel severe right ankle pain without any trigger events. Although treatment, including physiotherapy and NSAID administration, was initiated in another clinic, the pain in his ankle progressively worsened and he demonstrated gait disturbance. A physical examination revealed redness, swelling, and remarkable tenderness around his ankle. Severe pain and hyperalgesia also resulted in the disturbance of ankle motion and weight-bearing. Radiographs showed marked regional osteoporotic changes in the distal tibia and fibula at his right ankle compared with his left ankle (Figure 1A). Bone scintigraphy with ^99 m^Tc-methylene diphosphonate revealed a marked increase in radioactivity in the bones around his ankle (Figure 1B). These clinical findings were consistent with CRPS type I (CRPS I) according to criteria of the International Association for the Study of Pain [2]. His lumbar bone mineral density was 0.904 g/cm^2^, which is more than 80% of the Japanese young adult mean (YAM). Laboratory tests showed urinary crosslinked N-telopeptides of type I collagen (NTX), a bone resorption marker, to be 37.6 nmol bone collagen equivalent/mmol·Cr (nMBCE/mM·CR) (normal range, 9.3 to 54.3) and an alkaline phosphatase (ALP) level of 215 U/L (normal range, 110 to 370). Serum calcium (9.1 mg/dL; normal range 8.4 to 10.4), serum phosphate (2.9 mg/dL; normal range, 2.5 to 4.5), white blood cell count (5600/μL; normal range, 3900 to 9800), and C-reactive protein (< 0.1 mg/dL; normal range, < 0.3) were all at normal levels.

**Figure 2:**
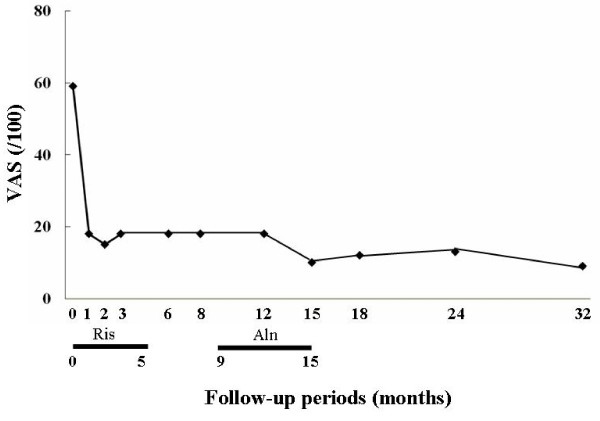
**Change in bone pain (visual analog scale, or VAS)**. The reduction in VAS score after the oral administration of low doses of bisphosphonate. Aln, duration of treatment with alendronate at 35 mg per week; Ris, duration of treatment with risedronate at 2.5 mg per day; VAS, visual analog scale (/100 mm).

**Figure 3:**
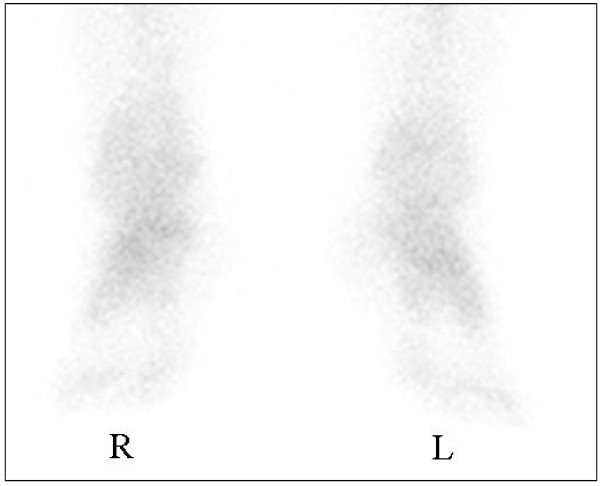
**Bone scintigraphs of the both ankles after treatment**. The previously increased radioactivity (Figure 1B) in the right ankle was markedly reduced, and findings were comparable to those of the normal, left ankle at 12 months after the start of bisphosphonate treatment.

**Figure 4:**
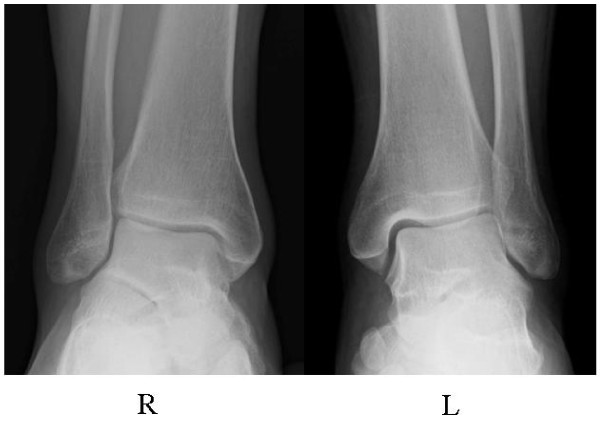
**Radiographs of the both ankles after treatment**. The regional osteoporotic change in the right ankle (Figure 1A) completely returned to normal, and findings were comparable to those of the left ankle at 32 months after the start of bisphosphonate treatment.

In accordance with the diagnosis of CRPS I accompanied by marked regional osteoporotic changes, oral administration of risedronate at 2.5 mg per day, the same dose used for the treatment of osteoporosis in Japan, was initiated to reduce the high bone turnover and ankle pain (VAS score of 59 out of 100). One month after the start of risedronate treatment, bone pain had fallen from a VAS score of 59 out of 100 to 18 out of 100, and we temporarily discontinued treatment at five months because of successful ankle pain reduction and the presence of epigastric pain. After nine months (four months after the discontinuation of treatment), we resumed bisphosphonate treatment with oral alendronate at 35 mg per week and the ankle pain decreased from a VAS score of 18 out of 100 to 10 out of 100 at 15 months (Figure 2). Bone scintigraphy at 12 months showed a marked reduction in radioactivity to a level comparable to that in the normal, left ankle (Figure 3). In addition, the effect of bisphosphonate on bone pain relief correlated with a reduction in NTX level, from 37.6 to 12.9 at eight months. On the basis of these results, treatment was discontinued at 15 months, and ankle pain relief lasted for 32 months (Figure 2). At the latest examination at 32 months, our patient had almost no pain (VAS score of 9 out of 100) and radiographic findings revealed that the regional osteoporotic change in his ankle had returned to normal, and findings were equivalent to those for his left ankle (Figure 4).

**Figure 5:**
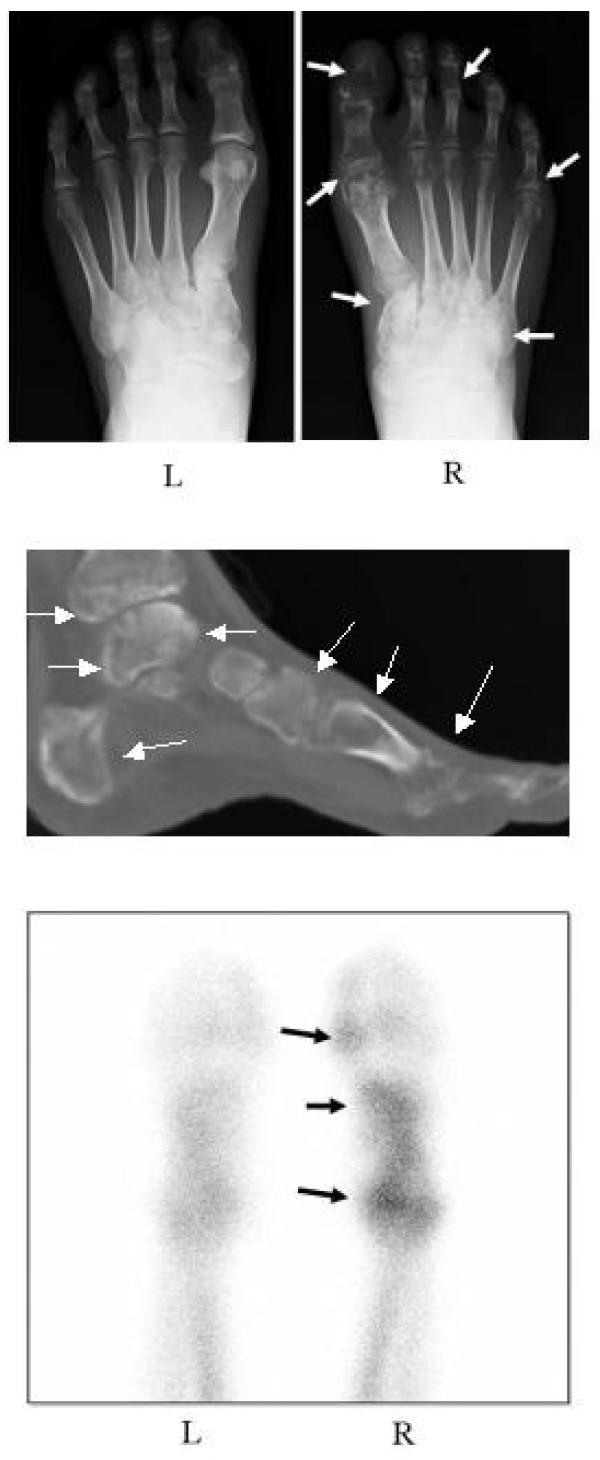
**Radiograph (A), reconstitution computed tomography (CT) image (B), and scintigraph (C) of the both feet before treatment**. The radiograph shows regional osteoporotic changes in the phalanges, metatarsals, and tarsals of the right foot compared with the normal, left foot (white arrow) (A). Reconstitution CT image exhibits the extensive osteoporotic changes in the foot and ankle (white arrow) (B). Bone scintigraphy with ^99 m^Tc-methylene diphosphonate shows a marked increase in radioactivity in the right foot (black arrow) (C).

A second 48-year-old Japanese man with CRPS was referred to our institute for treatment of severe right foot pain with a VAS score of 83 out of 100. About nine months earlier, he began to feel severe right foot pain without any trigger events. Although treatment, including physiotherapy and NSAID administration, was initiated in another clinic, the pain and swelling of his right foot progressively worsened, and he demonstrated gait disturbance. A physical examination revealed redness, swelling, and remarkable tenderness of his foot. Severe pain and hyperalgesia also resulted in the disturbance of weight-bearing. Radiographs showed regional osteoporotic changes in the phalanges, metatarsals, and tarsals of his right foot compared with his normal, left foot (Figure 5A). Reconstitution computed tomography also exhibited the extensive osteoporotic changes in his foot and ankle (Figure 5B). Bone scintigraphy with ^99 m^Tc-methylene diphosphonate revealed a marked increase in radioactivity in the bones of his foot (Figure 5C). These clinical findings were consistent with CRPS I according to the criteria [2] described for our first patient. Lumbar bone mineral density was 0.838 g/cm^2^, which is more than 80% of the YAM. Laboratory tests showed an NTX of 48.6 nMBCE/mM·CR and normal values of ALP (242 U/L), serum calcium (9.6 mg/dL), serum phosphate (3.1 mg/dL), white blood cell count (7100/μL), and C-reactive protein (< 0.1 mg/dL).

**Figure 6:**
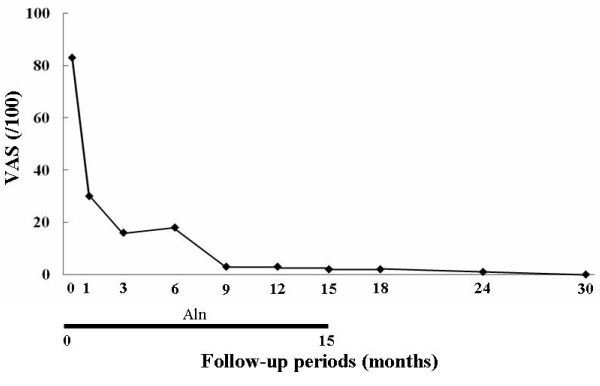
**Changes in bone pain (visual analog scale, or VAS)**. The reduction in VAS score after the oral administration of low doses of bisphosphonate. Aln, duration of treatment with alendronate at 35 mg per week; VAS, visual analog scale (/100 mm).

**Figure 7:**
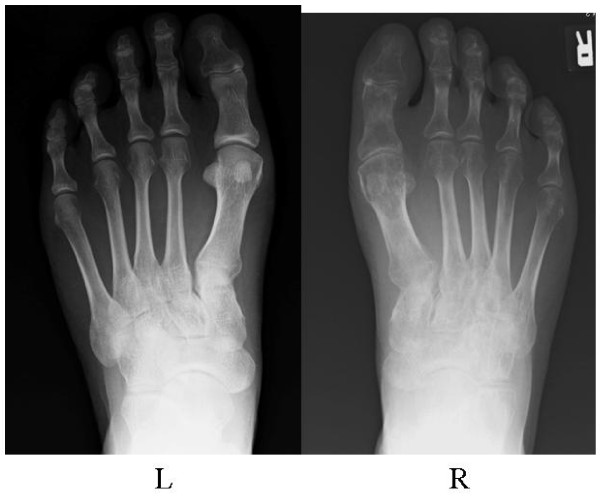
**Radiographs of the both feet after treatment**. The regional osteoporotic changes in the right foot (Figure 5A) were markedly improved at 32 months after the start of bisphosphonate treatment.

In accordance with the diagnosis of CRPS I accompanied by marked regional osteoporotic findings together with the successful treatment of our first patient, weekly oral administration of alendronate at 35 mg per week, the same dose used for the treatment of osteoporosis in Japan, was initiated to reduce the high bone turnover and foot pain (VAS score of 83 out of 100). One month after the start of alendronate treatment, bone pain had fallen from a VAS score of 83 out of 100 to 30 out of 100 and was further reduced to 18 out of 100 at three months and 3 out of 100 at nine months. The treatment was discontinued at 15 months because of successful pain reduction, and the pain relief lasted for 30 months without further alendoronate administration (Figure 6). The bone pain relief correlated with a decrease in NTX values: 12.0 at 15 months and 14.0 at 24 months. Our patient rejected follow-up bone scintigraphy because he experienced no symptoms. At the latest examination at 30 months, he had no pain (VAS score of 0 out of 100) and the radiographic findings revealed marked improvement in regional osteoporotic changes (Figure 7).

## Discussion

CRPS I is difficult to treat, despite the various methods that have been tried [3-5], and the therapeutic use of various drugs has been reported to be effective in some studies and useless in others [3,4,7]. Pathophysiologically, CRPS reveals enhanced regional bone resorption and high bone turnover, and so several reports have indicated that administration of bisphosphonate results in a significant reduction in pain [8-11,13,14]. However, in these studies, the method of administration was intravenous or in high oral doses (alendronate, 40 mg per day). Recent studies have shown that intravenous or high-dose bisphosphonate therapy increases the incidence of severe side effects, such as bisphosphonate-related osteonecrosis of the jaw [15] or severely suppressed bone turnover [16,17]. It was, therefore, considered ideal if low-dose bisphosphonate treatment could result in similar improvements in CRPS symptoms. In this report, we presented two patients who had CRPS I and who demonstrated marked pain relief and improvements in regional osteoporotic change in the foot or ankle as a result of the low-dose oral administration of bisphosphonate (risedronate at 2.5 mg per day or alendronate at 35 mg per week) at doses equivalent to those used for the treatment of osteoporosis in Japan. Also, in both cases, the ameliorative effects have lasted more than one year, even after the administration of bisphosphonate was discontinued. We administered the bisphosphonate as a daily risedronate or weekly alendronate dose, depending on epigastric symptoms and patient preference. To the best of our knowledge, few reports have indicated that a low dose of oral bisphosphonate has any efficacy in the treatment of CRPS I, particularly if the follow-up period after the discontinuation of treatment was more than one year.

The etiology of CRPS I varies, and several studies have indicated that most cases of CRPS I are caused by secondary etiologies such as trauma and diabetes [10,11]. Manicourt and colleagues [11] showed that traumatic events triggered CRPS I in most of their cases and that the pain associated with the disease was due not only to enhanced bone turnover but also to the production of proinflammatory cytokines and various neuropeptides from sustained peripheral nerve injury as a post-traumatic event [11,18,19]. The value of bisphosphonates in the treatment of CRPS I disease did not, therefore, seem great. However, in regard to the idiopathic cases of CRPS I presented here, bisphosphonate treatment markedly and rapidly improved the severe bone pain and afforded a concomitant reduction in bone turnover in the foot and ankle. It was previously recognized that bone disorders with increased osteoclastic bone resorption, such as Paget disease, are associated with bone pain [20], and the osteoclastic bone resorption was suggested to be critical to the pain, and the inflammation occurred adjacent to bone through an activation of the acid-sensing receptors through creation of acidosis by the osteoclasts [21]. In these cases, bisphosphonates, inhibitors of osteoclast activity, were shown to reduce bone pain. Thus, in our idiopathic cases, accelerated and enhanced bone resorption and turnover in the foot or ankle might have played a dominant pathophysiological role in the development of CRPS I rather than peripheral nerve disorder as a post-traumatic injury.

This report has a limitation. We cannot deny the possibility that the observed improvement of pain and osteoporotic changes was a consequence of spontaneous amelioration of the disease. However, in regard to the immediate improvement of pain and regional osteoporosis change after the initiation of bisphosphonate treatment and the ineffectiveness of the previous treatment (including physiotherapy and NSAID administration for about six months), the improvement could be considered the effect of bisphosphonate. Thus, we believe that low-dose oral administration of bisphosphonate is worth considering for the treatment of idiopathic CRPS I accompanied by high regional bone turnover.

## Conclusions

In two patients with CRPS I, the oral administration of low-dose bisphosphonate resulted in an improvement in severe pain and regional osteoporotic findings in the foot or ankle. We speculate that a low dose of oral bisphosphonate might also be effective for the reduction in pain in cases of idiopathic CRPS I, particularly when accompanied by regional osteoporotic changes.

## Abbreviations

ALP: alkaline phosphatase; CRPS: complex regional pain syndrome; CRPS I: complex regional pain syndrome type I; NSAID: nonsteroidal anti-inflammatory drug; NTX: N-telopeptides of type I collagen; VAS: visual analog scale; YAM: young adult mean.

## Consent

Written informed consent was obtained from the patients for publication of this case report and any accompanying images. A copy of the written consent is available for review by the Editor-in-Chief of this journal.

## Competing interests

The authors declare that they have no competing interests.

## Authors' contributions

YA, JT, and TW performed the bisphosphonate treatment and several examinations of the patients and carried out the follow-up of the patients for more than 30 months. KI performed the bisphosphonate treatment and several examinations of the patients, carried out the follow-up of the patients for more than 30 months, conceived of the study, participated in its design and coordination, and helped to draft the manuscript. TY conceived of the study, participated in its design and coordination, and helped to draft the manuscript. All authors read and approved the final manuscript.
